# From the Editors – Future Directions to Strengthen the Emergency Department Safety Net

**DOI:** 10.5811/westjem.60719

**Published:** 2023-08-08

**Authors:** Rama A. Salhi, Wendy L. Macias-Konstantopoulos, Caitlin R. Ryus

**Affiliations:** * Massachusetts General Hospital, Department of Emergency Medicine, Boston, Massachusetts; ** Yale School of Medicine, Department of Emergency Medicine, New Haven, Connecticut

Emergency medicine as a specialty prides itself on being the safety net for the communities we serve. Always open, we provide care to all comers, regardless of insurance status or ability to pay, often on the worst days of their lives. Over time, we have come to appreciate that this can mean fractured limbs, cardiac arrest, or a cancer diagnosis; but just as frequently we care for individuals who are suffering the ramifications of the innumerable ways in which our system has failed them. This includes poverty, structural racism, lack of housing, food insecurity, community violence, and widely disparate incarceration rates.^1^^–^^3^

We acknowledge several fundamental truths:

1. Discrimination by age, race, gender identity, sexual orientation, housing status, psychiatric illness (and the list goes on) exists.
2. Our patients, colleagues, and communities grapple with discrimination and its health ramifications every day.
3. Implicit biases and discriminatory practices are not static. We continue to see the impact of past practices, and our current decisions will impact many decades to come.
4. Emergency clinicians are uniquely positioned and privileged to contribute to advancing equity.

As we strive to understand more fully the health impact of these social inequities and injustices, our clinical specialty has seen the rapid expansion of social emergency medicine. Increasingly recognized as a subspecialty, social emergency medicine has established a growing foundation of knowledge replete with peer-reviewed literature, academic journal special issues, textbooks, research and educational conferences, fellowships, and representation within our professional societies.^4^^–^^6^ The burgeoning multidisciplinary work of social emergency medicine is a testament to the nuance with which we must understand social and structural determinants of health. This level of nuance is required to even begin to understand our role as emergency physicians, administrators, educators, researchers, and ultimately advocates in their solutions.

Despite these advances, the body of literature that constitutes the foundation of social emergency medicine is still being built. Many of the social determinants of health that we seek to address can be difficult to quantitatively study due to small or inaccessible populations, discrimination and mistrust, or failure to screen and identify. Here is where we find the value of case studies, qualitative studies, hypothesis-generating studies, and narrative reviews. Showcased in this *Western Journal of Emergency Medicine* special issue are studies by authors who endeavored to contribute to our growing foundation of knowledge and cover a broad range of topics.

As emergency physicians, we bear witness daily to the health impacts of social inequities and injustices on the individuals and communities we serve. Although the structural solutions needed to address our most vexing social problems may not lie squarely within our control as a specialty, we have both the responsibility and the opportunity to create, inform, and participate in change. Systematically investigating the compounding health effects of complex and interrelated social problems allows us to fulfill our promise to our communities: No matter who you are, no matter your problem, we are here 24 hours a day, 365 days a year.
